# Anti-inflammatory effects of sargachromenol-rich ethanolic extract of *Myagropsis myagroides* on lipopolysaccharide-stimulated BV-2 cells

**DOI:** 10.1186/1472-6882-14-231

**Published:** 2014-07-09

**Authors:** Sunghee Kim, Min-Sup Lee, Bonggi Lee, Wi-Gyeong Gwon, Eun-Ji Joung, Na-Young Yoon, Hyeung-Rak Kim

**Affiliations:** 1 Department of Food Science and Nutrition, Pukyong National University, Yongso-ro, Nam-gu, Busan 608-737, South Korea; 2 National Institute of Child Health and Human Development, National Institutes of Health, Bethesda, MD 20892, USA; 3 Food and Safety Research Division, National Fisheries Research and Development Institute, 216, Gijanghaean-ro, Gijang-eup, Gijang-gun, Busan 619-705, South Korea; 4 Institute of Fisheries Sciences, Pukyong National University, Ilgwang-ro, Ilgwang-myeon, Gijang-gun, Busan 619-911, South Korea

**Keywords:** *Myagropsis myagroides*, Sargachromenol, Pro-inflammatory cytokine, Microglia, Nuclear factor-κB, Neuroinflammation

## Abstract

**Background:**

Excessive pro-inflammatory cytokine production from activated microglia contributes to neurodegenerative diseases, thus, microglial inactivation may delay the progress of neurodegeneration by attenuating the neuroinflammation. Among 5 selected brown algae, we found the highest antioxidant and anti-neuroinflammatory activities from *Myagropsis myagroides* ethanolic extract (MME) in lipopolysaccharide (LPS)-stimulated BV-2 cells.

**Methods:**

The levels of nitric oxide (NO), prostaglandin E_2_ (PGE_2_), and pro-inflammatory cytokines were measured by Griess assay and enzyme linked immunesorbent assay. The levels of inducible nitric oxide synthase (iNOS), cyclooxygenase-2 (COX-2), mitogen-activated protein kinases (MAPKs), and Akt were measured using Western blot. Nuclear translocation and transcriptional activation of nuclear factor-κB (NF-κB) were determined by immunefluorescence and reporter gene assay, respectively.

**Results:**

MME inhibited the expression of iNOS and COX-2 at mRNA and protein levels, resulting in reduction of NO and PGE_2_ production. As a result, pro-inflammatory cytokines were reduced by MME. MME also inhibited the activation and translocation of NF-κB by preventing inhibitor κB-α (IκB-α) degradation. Moreover, MME inhibited the phosphorylation of extracellular signal regulated kinases (ERKs) and c-Jun N-terminal kinases (JNKs). Main anti-inflammatory compound in MME was identified as sargachromenol by NMR spectroscopy.

**Conclusions:**

These results indicate that the anti-inflammatory effect of sargachromenol-rich MME on LPS-stimulated microglia is mainly regulated by the inhibition of IκB-α/NF-κB and ERK/JNK pathways.

## Background

Microglia, a macrophage-like cells in the brain, play a pivotal role in the innate immune response in the central nervous system. Microglia are activated by the broad spectrum of stimuli such as lipopolysaccharide (LPS), interferon-γ, or β-amyloid [1,2]. Activated microglia produce various neurotoxic factors including inflammatory mediators such as nitric oxide (NO) and prostaglandin E_2_ (PGE_2_), and pro-inflammatory cytokines such as tumor necrosis factor-α (TNF-α), interleukin (IL)-1β, and IL-6 [3,4]. Pathogenic roles of inflammatory mediators and cytokines have been implicated in various inflammatory and neurodegenerative diseases, including Alzheimer’s disease, Parkinson’s disease, trauma, multiple sclerosis, and cerebral ischemia [5,6].Therefore, the modulation of microglial activation is important for the prevention or relief of neuroinflammation.

The induction of inflammatory proteins and pro-inflammatory cytokines are primarily controlled at transcriptional level [7]. Transcriptional induction of inducible nitric oxide synthase (iNOS) and cyclooxygenase-2 (COX-2) is largely dependent on cooperative activities of multiple transcription factors, including nuclear factor-κB (NF-κB) and activator protein 1 which act on cognate cis-acting elements in the iNOS or COX-2 promoter [7,8]. NF-κB plays key roles in early stage of immune and inflammatory responses as well as cell survival [9,10]. In unstimulated conditions, NF-κB bound with inhibitory kappaB-α (IκB-α) is located in the cytoplasm as an inactive complex. Exposure to LPS stimulates phosphorylation, ubiquitination, and degradation of IκB-α, resulting in nuclear translocation of NF-κB by dissociation of NF-κB-IκB-α complex for the transcription of target genes [11]. The activation of NF-κB is also regulated by cellular kinases such as mitogen-activated protein kinases (MAPKs) [12]. The MAPKs such as extracellular signal-regulated kinase (ERK), p38 MAPK, and c-Jun NH_2_-terminal kinase (JNK) have been involved in the transcriptional regulations of inflammatory genes [12,13].

Marine macroalgae have been used as a healthy diet in East Asia for centuries. Recently, various studies revealed that their constituents such as phlorotannins and pigments showed diverse biological activities including antioxidation [14-16] and anti-inflammation [15,17-20]. *Myagropsis myagroides*, growing subtidal zone of the coast of East Asia, belongs to the family Sargassaceae in Phaeophyta. It shows the anti-inflammatory activity and its active compound was tentatively identified as phlorofucofuroeckol B by NMR spectroscopy and 6,6’-bieckol [21,22]. However, we found that *M. myagroides* ethanolic extract (MME) showed higher anti-inflammatory activity than phlorofucofuroeckol B and 6,6’-bieckol and the isolated compound was identified as sargachromenol. Biological activities of sargachromenol were limited to anti-photoaging activity [23], anti-cholinesterase activity [24], and neuronal growth factor [25]. To our knowledge, no previous study has been reported on the anti-inflammatory activity of sargachromenol-rich MME in LPS-treated BV-2 cells. BV-2 cells, derived from primary mouse microglia cells, are considered as a reasonable model for *in vitro* pharmacological studies, since their response to LPS showed a similar pattern to primary microglia *in vivo* based on transcriptome and proteome analysis [26]. With respective to neurodegeneration studies, activated BV-2 cells by LPS secret pro-inflammatory cytokines, which have been shown to promote neuronal injury at high level [27]. This led us to evaluate the inhibitory effect of MME on inflammation using BV-2 cells, and we further investigated the possible molecular mechanisms underlying its anti-inflammatory action on cultured BV-2 cells.

## Methods

### Algae materials and preparation of ethanolic extracts

*Myagropsis myagroides* (MBRB0078-TC10499)*, Undaria pinnatifida* (MBRB0049-TC9322)*, Saccharina japonica* (MBRM0094-TC11278)*, Sargassum horneri* (MBRB0037-TC9244)*,* and *S. fulvellum* (MBRB00112-TC7337) were collected along the coast of Busan, South Korea from January to August 2012. Taxonomic identification of the collected seaweeds was authenticated by an agal taxonomist (C.G. Choi), at the Department of Ecological Engineering, Pukyong National University, South Korea. Voucher specimens were deposited in the Marine Brown Algae Resources Bank, South Korea. The collected seaweeds were sun-dried for 3 days and ground with hammer mill. Each dried powder (100 g) was extracted three times with 500 mL of ethanol (95%, v/v) for 3 h at 70°C. The combined extracts were concentrated using a rotary vacuum evaporator (Eyela, Tokyo, Japan) at 40°C and lyophilized to obtain the ethanolic extracts of seaweed.

### Chemicals

Cell culture medium and all the other materials required for cell culture were purchased from Gibco BRL Life Technologies (Grand Island, NY, USA). LPS (*Escherichia coli* O55:B5), dimethyl sulfoxide (DMSO), bovine serum albumin (BSA), and the specific protein kinase inhibitors (PD98059 and SP600125) were purchased from Sigma Chemical Co. (St. Louis, MO, USA). CellTiter^96^ AQ_ueous_ One Solution Cell Proliferation assay kit, dual-luciferase assay system, murine NF-κB promoter/luciferase DNA, pRL-TK DNA, and moloney murine leukemia virus (M-MLV) reverse transcriptase were obtained from Promega (Madison, WI, USA). Enzyme-linked immunosorbent assay (ELISA) kits for TNF-α, IL-1β, and IL-6 were obtained from eBioscience (San Diego, CA, USA) and PGE_2_ ELISA kit was purchased from R&D Systems (Minneapolis, MN, USA). Primary and secondary antibodies were purchased from Cell Signaling Biotechnology (Danvers, MA, USA) and Santa Cruz Biotechnology (Santa Cruz, CA, USA), respectively. 4’,6-Diamidino-2-phenylindole (DAPI), Lipofectamine Plus Reagent, TRIzol, and Alexa Fluor® 488-conjugated secondary antibody were purchased from Invitrogen (Carlsbad, CA, USA). The enhanced chemiluminescence (ECL) detection kit was purchased from GE Healthcare Life Sciences (Piscataway, NJ, USA).

### Measurement of total phenolic content

Total phenolic content was measured according to the method of Koivikko et al. (2005) [28]. In brief, diluted sample 0.5 mL was mixed with 0.5 mL of 1 N Folin-Ciacalteu solution and incubated at 37°C. After 5 min, 1.0 mL of 20% sodium carbonate was added and the mixture was incubated for 30 min. The absorbance was measured at 730 nm, and total phenolic content was calculated using a phloroglucinol (Sigma Chemical Co.) as a standard.

### Cell culture and viability assay

Murine BV-2 microglial cell lines were maintained in Dulbecco’s Modified Eagle’s Medium (DMEM) supplemented with 10% fetal bovine serum (FBS), penicillin (100 units/mL), and streptomycin sulfate (100 μg/mL) in a humidified atmosphere of 5% CO_2_. Cell viability was determined by 3-(4,5-dimethylthiazol-2-yl)-5-(3-carboxymethoxyphenyl)-2-(4-sulfophenyl)-2H-tetrazolium (MTS) assay using CellTiter^96^ AQ_ueous_ One Solution Cell Proliferation assay kit according to the manufacturer’s manual. Cells were inoculated at a density of 3 × 10^5^ cells into 96-well plates and cultured at 37°C for 24 h. Cells were then treated with LPS (1 μg/mL) in the presence or absence of MME in different concentration for 24 h. The final concentration of DMSO was less than 0.1% in the cell culture medium. The culture medium was removed and replaced by 95 μL of fresh culture medium and 5 μL of MTS solution. After 1 h, the absorbance at 490 nm was measured using a microplate reader (Glomax Multi Detection System, Promega).

### Measurement of intracellular ROS

The intracellular ROS scavenging activity of the sample was measured using the fluorescent probe 2’,7’-dichlorodihydrofluorescin diacetate (DCFH-DA). The cells were incubated with different concentrations of extracts in the absence or presence of LPS (1 μg/mL) for 2 h. Harvested cells by trypsin-EDTA solution [0.05% trypsin and 0.02% EDTA in phosphate buffered saline (PBS)] were washed twice with PBS and treated with 20 μM DCFH-DA for 30 min at 37°C. The fluorescence intensity was measured at excitation wavelength of 485 nm and emission wavelength of 528 nm using a fluorescence microplate reader (Dual Scanning SPECTRAmax, Molecular Devices Co., Sunnyvale, CA, USA). Relative ROS level was adjusted with protein concentration of cell lysates by BCA protein assay (Pierce Biotechnology, Rockford, IL, USA).

### Measurements of NO, PGE_2_ and pro-inflammatory cytokines

Cells (5 × 10^4^ cells/well) were pretreated with MME (0–25 μg/mL) for 2 h prior to LPS treatment for 24 h. After treatment of LPS, cultured media of BV-2 cells were collected and stored at -72°C until tested. For the measurement of NO, 100 μL of culture *s*upernatant was mixed with the same volume of Griess reagent (0.1% naphthylethylenediamine dihydrochloride and 1% sulfanilamide in 5% phosphoric acid) and incubated at room temperature for 10 min. Absorbance of the mixture was measured with a microplate reader at 540 nm. Levels of PGE_2_, TNF-α, IL-1β, and IL-6 in culture media from each group were quantitatively determined by ELISA kit according to the manufacturer’s instructions.

### Western blot analysis

Proteins (30 μg) were separated by sodium dodecyl sulfate-polyacrylamide gel electrophoresis and transferred onto the nitrocellulose membranes. The membranes were washed with Tris-buffered saline (10 mM Tris–HCl, 150 mM NaCl, pH 7.5) supplemented with 0.05% Tween 20 (TBST) followed by blocking with TBST containing 5% non-fat dried milk. The membranes were incubated overnight with primary antibodies. After washing three times with TBST, the membranes were then exposed to secondary antibodies coupled to horseradish peroxidase for 2 h at room temperature. The membranes were washed three times with TBST at room temperature. Immunoreactivities were detected by ECL reagents. Densitometric analysis of the data obtained from at least three independent experiments was performed using cooled CCD camera system EZ-Capture II (ATTO & Rise Co., Tokyo, Japan) and CS analyzer ver. 3.00 software (ATTO & Rise Co.).

### Reverse transcription-polymerase chain reaction (RT-PCR)

BV-2 cells plated in a 6-well cell culture plate at a density of 3.0 *×* 10^5^ cells*/*well were pretreated without or with MME for 2 h and then treated with LPS for 6 h. Total RNA from each group was isolated with the TRIzol reagent. Five micrograms of total RNA was used for reverse transcription using oligo-dT and M-MLV reverse transcriptase. PCR was carried out using the resulting cDNA as a template, with the following condition: 25 cycles of denaturation at 95°C for 30 s, annealing at 60°C for 30 s, and extension at 72°C for 30 s. Verification of PCR product of specific genes was established by their predicted sizes under ultraviolet light illuminator. The primer sequences were following: 5’-ACC ACT CGT ACT TGG GAT GC-3’ (sense), 5’-CAC CTT GGA GTT CAC CCA GT-3’ (antisense) for iNOS (accession no. NM_010927); 5’-TGG GCA AAG AAT GCA AAC AT-3’ (sense); 5’-CAG CAA ATC CTT GCT GTT CC-3’ (antisense) for COX-2 (accession no. NM_011198); 5’-GAC CCC TTC ATT GAC CTC AA-3’ (sense), 5’-CTT CTC CAT GGT GGT GAA GA-3’ (antisense) for glyceraldehyde 3-phosphate dehydrogenase (GAPDH) (accession no. NM_008084). GAPDH was used as an internal standard to evaluate relative expression of COX-2 and iNOS. Densitometric analysis of the data obtained from at least three independent experiments was performed using CS analyzer ver. 3.00 software.

### Immunofluorescence analysis

To analyze nuclear localization of NF-κB in BV-2 cells, cells were cultured on glass coverslips (SPL Lifesciences Co., Gyeonggi-do, Korea) in 24-well plates for 24 h. After preincubation with MME for 2 h, cells were stimulated with or without LPS (1 μg/mL) for 30 min. Cells were fixed in 4.0% paraformaldehyde in PBS for 15 min at room temperature, and then permeabilized with 0.5% Triton X-100 in PBS for 10 min. Permeabilized cells were washed with PBS and blocked with 3% BSA in PBS for 30 min. Thereafter, cells were incubated in an anti-NF-κB monoclonal antibody diluted in 3% BSA/PBS for 2 h, rinsed three times for 5 min with PBS, and incubated in Alexa Fluor® 488-conjugated secondary antibody diluted in 3% BSA/PBS for 1 h. Cells were stained with 2 μg/mL DAPI and viewed, and images were captured using an LSM700 laser scanning confocal microscope (Carl Zeiss, Oberkochen, Germany).

### Preparation of cytosolic and nuclear extracts

BV-2 cells plated in a 6-well cell culture plates at a density of 1 × 10^6^ cells per well were pretreated with or without MME for 2 h and then treated with LPS for 0.5 h. Cells were washed twice with ice-cold PBS, scraped in PBS and centrifuged at 13,000 *g* for 5 min at 4°C. Pellets were suspended in 180 μL of hypotonic buffer A [10 mM Tris–HCl (pH 7.4), 10 mM NaCl, 3 mM MgCl_2_, 0.02% NaN_3_, 0.5 mM DTT and 1 mM PMSF] on ice, and afterward, 20 μL of 5% Nonidet P-40 was added for 5 min. The mixture was centrifuged at 1,800 *g* for 5 min. Supernatant was collected as cytosolic extract. The pellets were washed with hypotonic buffer and resuspended in hypertonic buffer C [20 mM 4-(2-hydroxyethyl)-1-piperazineethanesulfonic acid (pH 7.4), 25% glycerol, 420 mM NaCl, 1.5 mM MgCl_2_, 0.2 mM EDTA, 0.02% NaN_3_, 0.5 mM DTT, and 1 mM PMSF] for 1 h on ice and centrifuged at 14,000 *g* for 10 min. The supernatant containing nuclear proteins was collected and stored at -72°C after determination of the protein concentration.

### NF-κB promoter/luciferase assay

Two micrograms of pNF-κB promoter/luciferase DNA along with 40 ng of control pRL-TK DNA was transiently transfected into 2.0 × 10^5^ BV-2 microglia cells per well in a six-well plate using Lipofectamine Plus reagents for 40 h. Cells were treated with MME for 2 h and stimulated with LPS (1 μg/mL) for 6 h. Luciferase activities of the cells were measured using dual-luciferase assay system according to the manufacturer’s instructions. Each transfection was performed in triplicate, and all experiments were repeated at least three times. The luciferase activity was normalized with luciferase activity of control pRL-TK.

### Isolation and identification of sargachromenol from MME

Aliquots of MME were dissolved in methanol and separated by Shimadzu high-performance liquid chromatography (HPLC) system with Luna RP-18 [Luna C18(2), 5 μm, 250 × 10 mm, Phenomenex, Torrence, CA, USA]. The separation of MME was conducted using 100% methanol (solvent A) and 0.1% formic acid in water (solvent B) as a mobile phase. The elution profile consisted of a linear gradient from A/B (78/22) to A/B (95/5) in 90 min and hold for 10 min and then re-equilibration of the column with A/B (78/22) for 18 min. The flow rate was 3.5 mL/min at 35°C oven temperature and detection was performed at 270 nm. Fractions were collected and assessed for the ability to inhibit NO secretion using LPS-stimulated BV-2 cells. The isolated compound (10 mg) was dissolved in 0.6 mL of CDCl_3_ and used for ^1^H- and ^13^C-NMR spectroscopy. NMR spectra were obtained by Fourier transform NMR JNM ECP-400 (JEOL, Tokyo, Japan). The chemical structure of the purified compound was identified by comparing its data with literature [24].

### Statistical analysis

Data were expressed as the means ± SDs of at least three independent experiments unless otherwise indicated. Data were analyzed using one-way analysis of variance (ANOVA), followed by each pair of Student’s *t*-tests for multiple comparisons. Differences with a value of *p* < 0.05 were considered statistically significant. All analyses were performed using SPSS for Windows, version 10.07 (SPSS, Chicago, IL, USA).

## Results

### Total phenolic contents and inhibitory activities of the seaweed extracts on NO and ROS production

*M. myagroides* and *S. horneri* showed higher yield of ethanolic extract than the other seaweeds and *M. myagroides* showed highest phenolic contents (Table 1). Since phenolic compounds are commonly found in plants and have been reported to have diverse biological activities including antioxidant and anti-inflammatory activities, we determined inhibitory activities of ROS and NO production of the extracts. *M. myagroides* showed highest antioxidant activity expressed as ROS scavenging activity (EC_50_, 112.4 ± 8.2 μg/mL) and anti-inflammatory activity expressed as the inhibition of NO production (EC_50_, 6.84 ± 0.78 μg/mL) in LPS-treated BV-2 cells. Due to strong anti-inflammatory activity and high phenolic contents, MME was chosen to investigate the anti-inflammatory properties and its underlying mechanisms.

**Table 1:** Phenolic contents, NO and ROS suppressive activities, and yields of ethanolic extracts from the selected brown seaweeds

	**Phenols**	**NO**^**1)**^	**ROS**^**2)**^	**Yield (%)**
	**(mg/g)**	**(EC**_**50**_**, μg/mL)**	**(EC**_**50**_**, μg/mL)**	
*Myagropsis myagroides*	40.4 ± 2.40	6.84 ± 0.78	112.4 ± 8.2	15.9 ± 2.1
*Sargassum horneri*	17.6 ± 1.48	> 100	134.2 ± 15.3	16.0 ± 1.1
*Sargassum fulvellum*	12.1 ± 0.71	53.1 ± 1.86	402.1 ± 34.2	13.4 ± 0.79
*Undaria pinnatifida*	9.46 ± 0.86	32.9 ± 3.24	240.3 ± 19.3	8.90 ± 0.81
*Saccharina japonica*	9.61 ± 0.03	43.9 ± 2.95	208.6 ± 16.2	13.4 ± 0.98
Sargachromenol		1.14 ± 0.11	8.31 ± 0.92	

All values are duplicated assays ± standard errors for three replicated extractions (i.e., n = 6)

EC_50_, half maximal effective concentration.

^1)^Inhibitory activity of NO production in LPS-treated BV-2 cells.

^2)^Inhibitory activity of ROS generation in LPS-treated BV-2 cells.

### MME inhibits NO and PGE_2_ production in LPS-stimulated BV-2 cells

**Figure 1:**
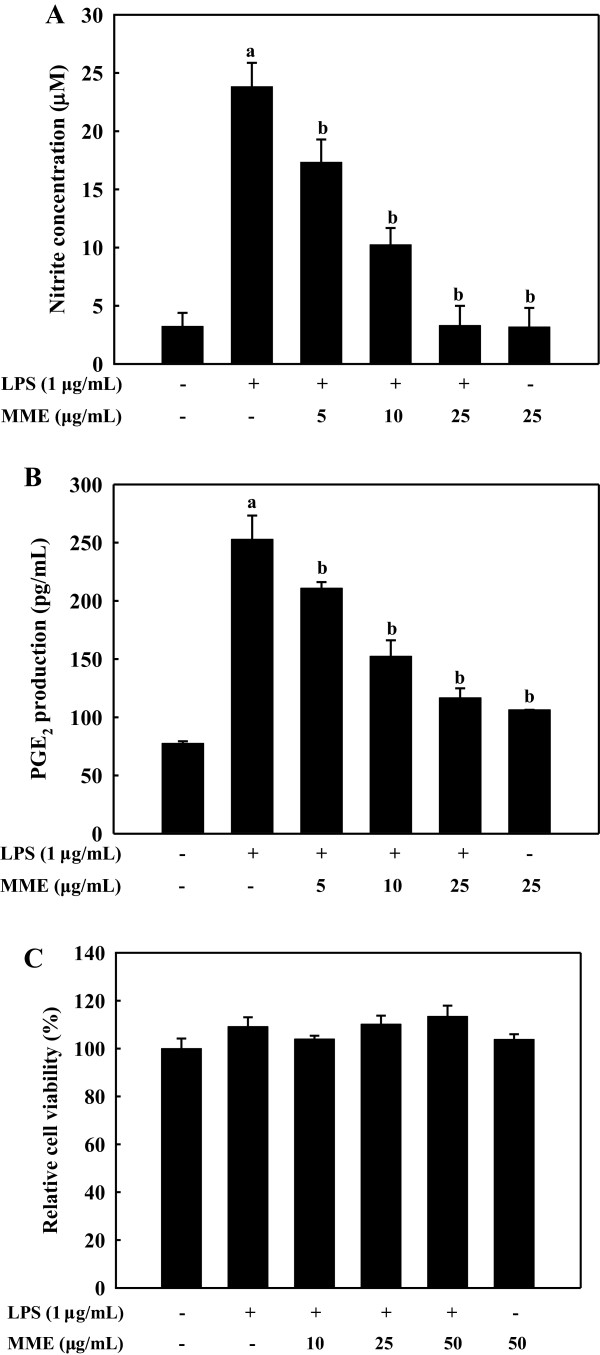
**Effect of MME on LPS-induced NO and PGE**_**2 **_**production in BV-2 cells.** BV-2 cells pretreated with different concentrations of MME for 2 h were stimulated with LPS (1 μg/mL) for 24 h. The treated culture media were used to assay the amount of NO **(A)** and PGE_2_ production **(B)**. BV-2 microglial cells were treated with various concentration of MME in the absence or presence of LPS (1 μg/mL). After 24 h, cell viability was measured by MTS assay **(C)**. Data are presented as means ± SDs of three independent experiments. ^a^*p* < 0.05 indicates significant differences compared to the non-treated group. ^b^*p* < 0.05 indicates significant differences compared to the LPS-only group.

To evaluate the effect of MME on LPS-induced production of inflammatory mediators including NO and PGE_2_, BV-2 cells were pretreated with 0–25 μg/mL MME for 2 h and stimulated with LPS for 24 h. NO production, measured as nitrite, was increased by LPS, however, MME significantly reduced NO levels in LPS-stimulated cells in a dose-dependent manner (*p* < 0.05, Figure 1A). Increased PGE_2_ production by LPS was also significantly suppressed by MME in a dose-dependent manner with EC_50_ value of 8.40 ± 1.08 μg/mL (Figure 1B). To exclude the possibility that the decreased NO and PGE_2_ levels were due to cell death, cytotoxicity of MME was determined by MTS assay. The result demonstrated that MME showed no cytotoxicity in BV-2 cells up to 50 μg/mL (Figure 1C). Thus, the inhibitory effects of MME on NO and PGE_2_ production were not due to cytotoxicity.

### MME inhibits iNOS and COX-2 expressions in LPS-stimulated BV-2 cells

**Figure 2:**
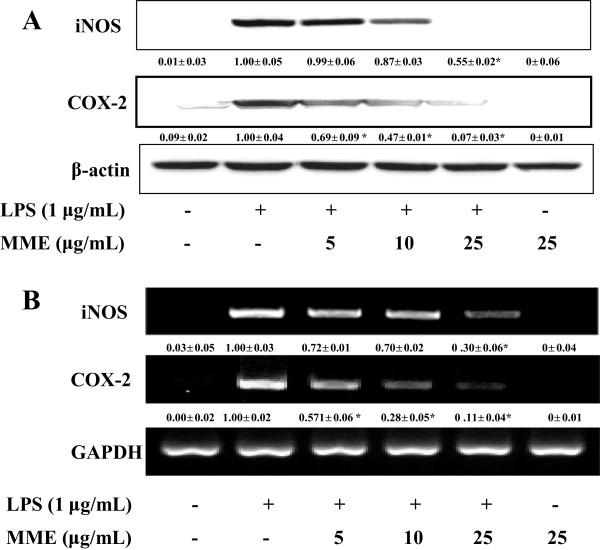
**Effect of MME on LPS-induced iNOS and COX-2 expressions in LPS-stimulated BV-2 cells. (A)** BV-2 cells were pretreated with different concentrations of MME for 2 h and stimulated with LPS (1 μg/mL) for 16 h. The expression of iNOS, COX-2, and β-actin protein was detected by Western blot using corresponding antibodies. **(B)** Cells pretreated with various concentrations of MME for 2 h were stimulated with LPS (1 μg/mL) for 6 h, and then total RNA was prepared for RT-PCR. The results presented are representative of three independent experiments. ^a^*p* < 0.05 indicates significant differences compared to the non-treated group. ^b^*p* < 0.05 indicates significant differences compared to the LPS-only group.

To investigate whether the inhibitory effects of MME on NO and PGE_2_ production are related to the regulation of the expression of iNOS and COX-2 proteins, respectively, BV-2 cells were pretreated with the indicated concentrations of MME for 2 h and then stimulated with LPS (1 μg/mL) for 16 h. LPS treatment induced a dramatic increase in iNOS and COX-2 proteins, as compared to the untreated control group (Figure 2A). Pretreatment of MME strongly inhibited the expression of iNOS and COX-2 proteins in a dose-dependent manner (*p* < 0.05). In addition to protein expression, MME inhibited iNOS and COX-2 mRNA expression in a dose-dependent manner in LPS-stimulated BV-2 cells (*p* < 0.05 Figure 2B). These results suggest that MME-mediated inhibition of NO and PGE_2_ production is associated with downregulation of iNOS and COX-2 genes at transcriptional level.

### MME inhibits LPS-induced TNF-α, IL-1β, and IL-6 secretion in BV-2 cells

**Figure 3:**
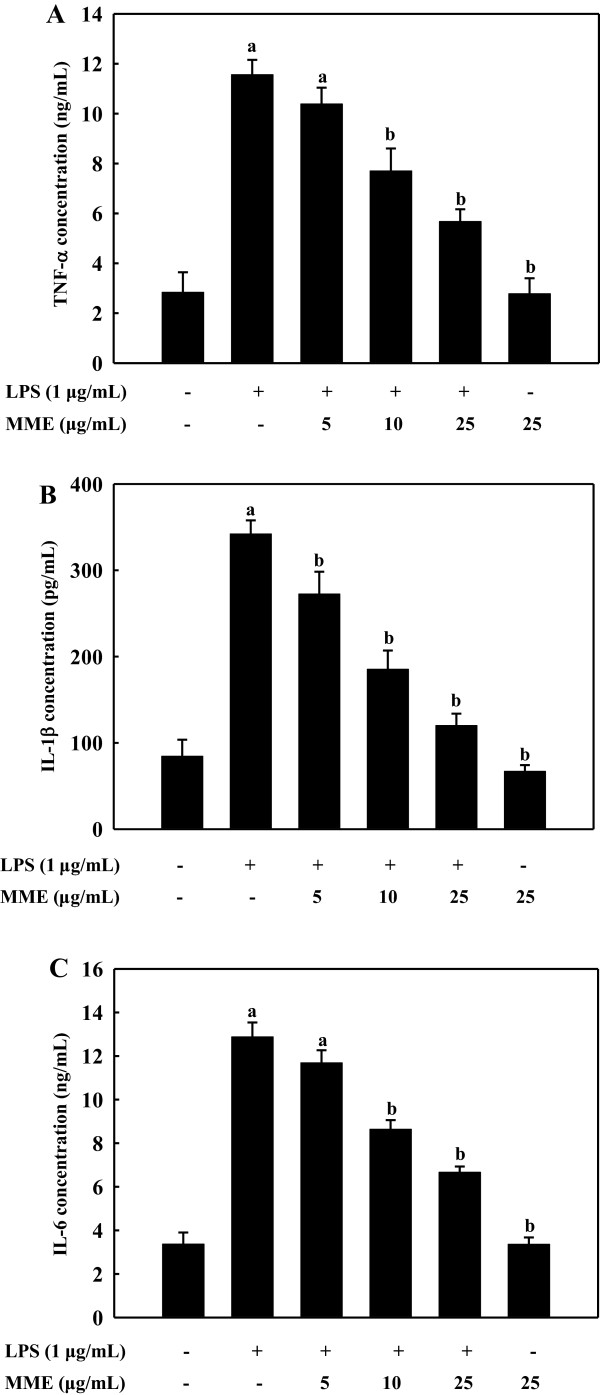
**Effects of MME on the secretion of pro-inflammatory cytokines in LPS-stimulated BV-2 cells.** BV-2 cells were treated with various concentrations of MME for 2 h, and then stimulated with LPS (1 μg/mL) for 24 h. TNF-α **(A)**, IL-1β **(B)**, and IL-6 **(C)** in the cultured media were measured by ELISA. Data are means ± SDs of three independent experiments. ^a^*p* < 0.05 indicates significant differences compared to the non-treated group. ^b^*p* < 0.05 indicates significant differences compared to the LPS-only group.

Since TNF-α, IL-1β, and IL-6 are early secreted pro-inflammatory cytokines and their levels are up-regulated in a variety of acute and chronic inflammatory diseases, we measured the effects of MME on the production of these cytokines in LPS-stimulated BV-2 cells. To reach detectable ranges of secreted cytokines in cultured media, LPS stimulations were extended up to 24 h. The stimulation of BV-2 cells with LPS induced remarkable increase in TNF-α (Figure 3A), IL-1β (Figure 3B), and IL-6 (Figure 3C). LPS-induced pro-inflammatory cytokines were significantly decreased by MME pretreatment in a dose-dependent manner (*p* < 0.05). The EC_50_ values of MME against TNF-α, IL-1β, and IL-6 were estimated to be 11.8 ± 1.2, 7.55 ± 0.5, and 14.1 ± 1.8 μg/mL, respectively. These results indicate that MME effectively suppressed LPS-induced TNF-α, IL-1β, and IL-6 production, indicating that MME inhibits the initial phase of the LPS-stimulated inflammatory response.

### MME inhibits degradation of IκB-α and translocation of NF-κB in LPS-stimulated BV-2 cells

**Figure 4:**
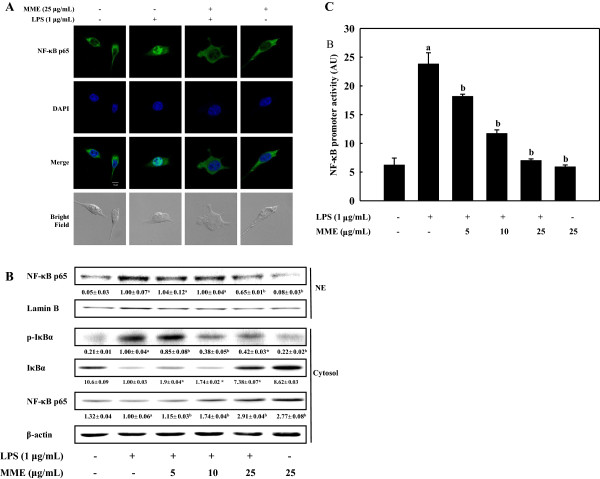
**Effect of MME on the translocation and activation of NF-κB in LPS-stimulated BV-2 cells. (A)** Cells were treated with and without MME for 2 h followed by LPS stimulation for 30 min. NF-κB/p65 subunits were probed by anti-NF-κB antibody and Alexa Fluor® 488-conjugated secondary antibody. The nuclei were stained by DAPI and the images were captured by confocal microscopy (×40). **(B)** Cells pretreated with different concentrations of MME for 2 h were stimulated with LPS for 30 min. Cytosolic and nuclear extracts were prepared and analyzed using Western blot by using corresponding antibodies. **(C)** Cells were co-transfected with 2 μg of NF-κB promoter-containing luciferase DNA along with 40 ng of control pRL-TK DNA for 40 h. Transfected cells were pretreated with various concentrations of MME for 2 h and then stimulated with LPS for 6 h. Cell lysates were prepared and used for reporter gene assay. Data are means ± SDs of three independent experiments. ^a^*p* < 0.05 indicates significant differences compared to the non-treated group. ^b^*p* < 0.05 indicates significant differences compared to the LPS-only group.

To determine transcriptional control of MME, we investigated the effects of MME on the nuclear translocation of NF-κB/p65 subunit using LPS-stimulated BV-2 cells. Confocal microscopic observations revealed that NF-κB/p65 was mainly located in the cytoplasm in unstimulated cells. After stimulation with LPS, most cytoplasmic p65 subunit was translocated into the nucleus, as shown in intense NF-κB/p65 staining in the nucleus (Figure 4A). However, the level of NF-κB/p65 in the nucleus was markedly reduced by pretreatment with MME. Considering the inhibitory effects of MME on LPS-induced NF-κB translocation into nucleus, we next analyzed the inhibitory effect of MME on LPS-stimulated phosphorylation of IκB-α and the translocation of NF-κB by Western blot. LPS treatment induced IκB-α phosphorylation, responsible for the activation of NF-κB, and MME pretreatment suppressed its phosphorylation and recovered the level of cytosolic IκB-α in a dose-dependent manner (Figure 4B). As a result of IκB-α phosphorylation, the increased nuclear NF-κB level by LPS was reduced by MME pretreatment in a dose-dependent manner. Additionally, we determined the effect of MME on the promoter activity of NF-κB in LPS-stimulated cells. The result suggests that MME pretreatment significantly inhibited LPS-induced NF-κB promoter activity of microglia in a dose-dependent manner (*p* < 0.05, Figure 4C). These results indicate that the MME-mediated inhibition of iNOS, COX-2, and pro-inflammatory cytokine productions were regulated by the inhibition of NF-κB activation in LPS-stimulated BV-2 cells.

### MME inhibits activation of JNK1/2 and ERK1/2 in BV-2 cells

**Figure 5:**
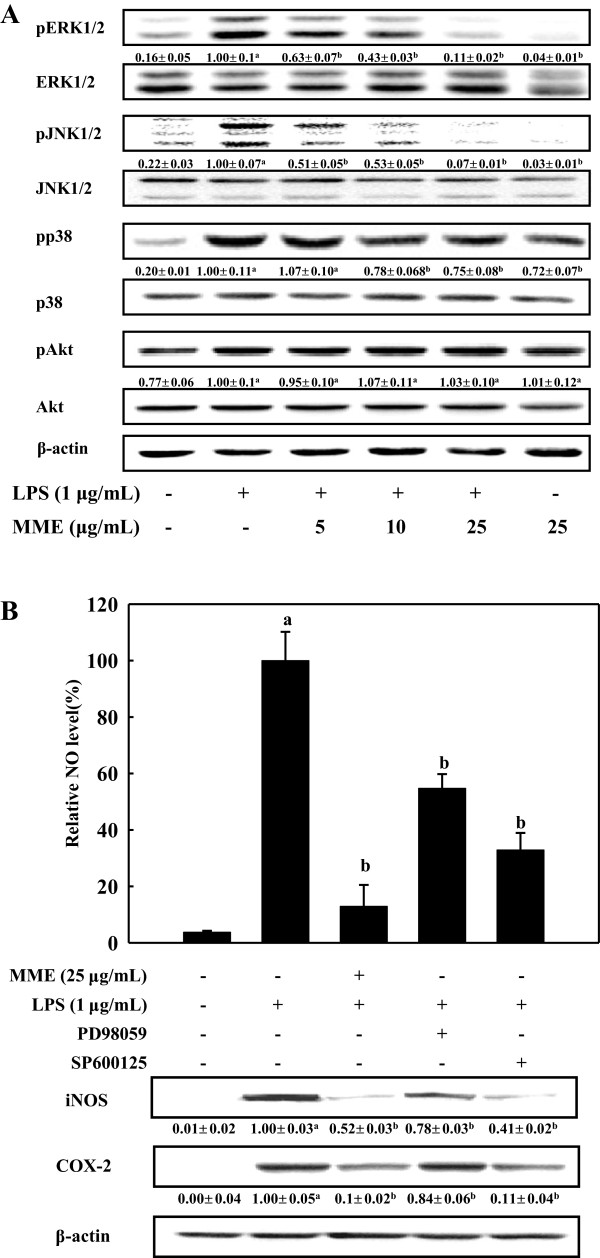
**Effects of MME on the phosphorylations of MAPKs and Akt in LPS-stimulated BV-2 cells. (A)** Cells were treated with various concentrations of MME for 2 h, and then stimulated with LPS (1 μg/mL) for 30 min. Whole cell lysates were prepared and analyzed by Western blot. Relative density ratios of pERK, pJNK, pp38 MAPK, and pAkt over ERK, JNK, p38 MAPK, and Akt, respectively, are shown below the blots. **(B)** Cell pretreated with indicated concentrations of MME or inhibitors for 2 h were stimulated with LPS (1 μg/mL) for 16 h. The culture media were collected for measuring NO, and total cell lysates were prepared for Western blot analysis. The results presented are representative of three independent experiments. Graphs represent means ± SDs of three independent experiments. ^a^*p* < 0.05 indicates significant differences compared to the non-treated group. ^b^*p* < 0.05 indicates significant differences compared to the LPS-only group.

To further investigate the molecular mechanisms of MME on the inhibition of NF-κB activation in LPS-stimulated BV-2 cells, we measured the inhibitory effect of MME on the phosphorylation of MAPKs, which are associated with the regulation of NF-κB pathway. As shown in Figure 5A, MME inhibited phosphorylation of ERK1/2 and JNK1/2 in a dose-dependent manner in LPS-stimulated cells, whereas there was no marked effect on the phosphorylation of Akt in BV-2 cells. In addition, MME slightly reduced p38 MAPK phosphorylation compared with ERK1/2 and JNK1/2 phosphorylation. To further confirm the association of these signaling proteins with the MME’s anti-inflammatory effect, we compared the production of NO and the protein levels of iNOS and COX-2 in the presence of MME, ERK inhibitor (PD98059) or JNK inhibitor (SP600125). As shown in Figure 5B, the levels of NO secretion from the LPS-stimulated BV-2 cells were remarkably inhibited by MME as well as by ERK or JNK inhibitors (*p* < 0.05). In addition, pretreatment with MME and SP600125 strongly inhibited LPS-induced NO production as well as iNOS and COX-2 production, whereas, pretreatment with PD98059 moderately inhibited the production of NO and inflammatory proteins. These results suggest that the additional characteristics of MME to regulated NF-κB pathway via blocking the phosphorylation of ERK1/2 and JNK1/2 proteins in response to LPS.

### Isolation of anti-inflammatory compound from MME

**Figure 6:**
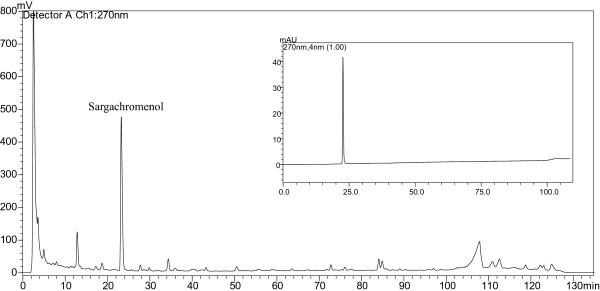
**Representative HPLC chromatogram of MME.** Aliquots of MME were separated by HPLC system and peak was identified by comparison of retention time to the corresponding authentic standard (inlet).

MME was further separated by reverse-phase column chromatography to identify anti-inflammatory activity (Figure 6). The peak indicated with sargachromenol showed a strong anti-inflammatory activity based on the inhibitory activity of NO production in LPS-treated BV-2 cells. The peak was collected by the repeated chromatographies and the chemical structure of the isolated compound was identified as sargachromenol from the comparison of its NMR spectra with the published spectral data. We obtained 4.5 mg of sargachromenol from 1 g of MME. The purified sargachromenol showed high anti-inflammatory activity in LPS-stimulated BV-2 cells (Table 1, EC_50_, 1.14 ± 0.11 μg/mL), indicating that sargachromenol is one of the major active compounds for anti-inflammatory activity of MME.

## Discussion

Brown seaweeds and their extracts are a well-known sources of antioxidation or anti-inflammation due to their appreciable amount of polyphenols and pigments. Although significant differences were observed in both total polyphenolic contents and antioxidant activities of extracts from various species, high correlation was found between total polyphenolic contents and their antioxidant capacity to scavenge ROS [29]. Polyphenolic compounds from marine algae may prevent inflammatory disorders, cancer, and diabetes which are associated with the regulation of free radicals generated in the cells [30,31]. In this regard, we analyzed antioxidant and anti-inflammatory activities of five representative brown algae along the southeastern coast of Korea. Among them, *M. myagroides* showed the highest phenolic contents and ROS scavenging activity as well as anti-inflammatory activity. High phenolic content in *M. myagroides* may participate in the inhibition of NO production in LPS-treated BV-2 cells. As anti-inflammatory activities from *M. myagroides*, fucoxanthin [32], fatty acid [33], 6,6-bieckol [22], and phlorofucofuroeckol B [21] have shown potent activities in macrophage or microglial cells. However, anti-inflammatory activity of MME in this study may not be due to fucoxanthin, fatty acid or phlorotannins, since the peaks of those compounds were not detected in the chromatogram (Figure 6) [20,21]. Thus, we hypothesized strong anti-inflammatory compounds are contained in MME and we separated the components from MME using C_18_ column and isolated sargachromenol having strong anti-inflammatory activity (Table 1). Base on the inhibitory activity of NO production in LPS-stimulated BV-2 cells, sargachromenol might be a main anti-inflammatory compound in MME.

NO and PGE_2_ are crucial inflammatory and neurotoxic mediators. These inflammatory mediators are responsible for the harmful effects on brain diseases, including ischemia, Alzheimer’s disease, and neuronal death [34]. *In vitro* and *in vivo* studies have revealed that overproductions of NO and PGE_2_ by enhanced iNOS and COX-2 protein levels, are associated with central nervous injuries and diseases [6]. iNOS and COX-2 proteins have been over-expressed in microglial cells from the rodent brain treated with LPS [2]. In addition, iNOS and COX-2 inhibitors provide neuroprotective effects against LPS-induced neurotoxicity, suggesting NO and PGE_2_ have important roles in neurotoxicity [6,35]. In this regard, inhibition of inflammatory mediator production is considered as a key step in the control of neuroinflammatory diseases. In the present study, we demonstrated that MME inhibited productions of both NO and PGE_2_ in LPS-stimulated BV-2 cells (Figure 1). Moreover, we provide evidence that MME-mediated inhibition of NO and PGE_2_ production was the consequence of the suppression of both mRNA and protein levels of iNOS and COX-2 in LPS-stimulated BV-2 cells (Figure 2). Furthermore, we found that the suppression of COX-2 mRNA expression by MME was more marked than that of COX-2 protein in BV-2 cells, indicating that inhibition of PGE_2_ by MME is associated with downregulation of COX-2 at both transcriptional and translational levels in LPS-stimulated BV-2 cells. Thus, the present findings may address that MME has protective effects on neurodegenerative diseases induced by neuroinflammation.

Pro-inflammatory cytokines, including TNF-α, IL-1β, and IL-6 are small secreted proteins that regulate immunity and inflammation. Their production is increased in inflammatory states and they function by regulating the intensity and duration of the immune response [3]. TNF-*α* plays a central role in initiating and regulating the cytokine signaling cascade during an inflammatory response in neuronal cells. In inflammatory disease states, TNF-*α* along with other pro-inflammatory mediators and neurotoxic substances is predominantly produced by activated microglia [4]. IL-1β is an important initiator of the immune response, playing a key role in the onset and development of a complex hormonal and cellular inflammatory cascade. IL-6 is a multifunctional cytokine that plays an important role in host defense, with major regulatory effects upon the inflammatory response [36]. Excessive productions of these pro-inflammatory cytokines activate microglia and lead to neural cell deaths, resulting in the pathogenesis of several neurological and neurodegenerative disorders [4,6,37]. As an alternative chemoprevention of inflammatory diseases, natural compounds able to inhibit the production of pro-inflammatory cytokines may be attractive as anti-inflammatory agents and, for this reason, the inhibitory effects of phytochemicals on the production of pro-inflammatory cytokines have been intensively studied to develop anti-inflammatory agents for preventing inflammatory diseases [38]. In the present study, we have demonstrated that MME remarkably suppressed the secretions of TNF-α, IL-1β, and IL-6 in LPS-stimulated BV-2 cells (Figure 3). Thus, the present findings may further support the potential of MME as a neuroprotective by reducing inflammation.

NF-κB plays an important role in the regulation of cell survival and coordinates the expression of pro-inflammatory proteins and cytokines, including iNOS, COX-2, TNF-α, IL-1β, and IL-6 [8]. NF-κB is present as an inactive complex associated with an inhibitory subunit, IκB-α, in cytoplasm. Activation of NF-κB caused by LPS or pro-inflammatory cytokines leads to degradation of IκB-α and inducing translocation of NF-κB into nucleus [12]. Recently, we demonstrated that extract of brown algae including *Saccharina japonica*[19,20], *M. myagroides*[21,22], *Ecklonia stolonifera*[15], and *Sargassum fulvellum*[39] inhibited the activation of NF-κB signaling pathway through the blockade of proteolytic degradation of IκB-α. In this study, we observed that enhanced phosphorylation of IκB-α by LPS was reduced by MME treatment, suggesting that MME protected the proteolytic degradation of IκB-α (Figure 4B). Degradation of IκB-α involves its dissociation from the inactive complex, leading to activation of NF-κB in response to LPS, which is demonstrated by NF-κB promoter activity (Figure 4C). Moreover, the nuclear translocation of NF-κB was significantly inhibited by MME, supporting the inhibition of NF-κB activation by MME (Figure 4A). From these data, the MME-mediated down-expression of LPS-induced inflammatory mediators and cytokines in BV-2 cells is partially associated with the ability of MME to inhibit the IκB/NF-κB signaling pathway.

NF-κB activation is alternatively regulated by various cellular kinases including MAPKs and Akt, which are the groups of protein kinases to play key roles in inflammatory reactions [38,40]. MAPKs are involved in inflammatory signaling cascades and regulation of iNOS and COX-2 through the activation of NF-κB in LPS-stimulated immune cells [9,12,38]. Therefore, anti-inflammatory mechanisms are closely related to inhibition of MAPKs in stimulated BV-2 cells. In this study, we have specifically shown that MME inhibits the activation of ERKs and JNKs, but not Akt and little p38 MAPK, in response to LPS in BV-2 cells, suggesting that ERKs and JNKs are additional targets of MME. Although, hexane fraction of MME down-regulated the phosphorylation of MAPKs and Akt in LPS-stimulated RAW 264.7 cells [22], it is hard to conclude why MME did not inhibit the phosphorylation of p38 MAPK and Akt in LPS-stimulated BV-2 cells. To further confirm the involvement of ERK1/2 and JNK1/2 on the activation of NF-κB, NO production and iNOS and COX-2 expression were determined. The inhibitory level of NO production showed 45% in the PD98059 treated cells and 65% in the SP600125 treated cells. MME treatment showed higher inhibitory effect of NO production than both inhibitors, which indicates that MME inhibits both phosphorylation of ERK1/2 and JNK1/2 (Figure 5B). In addition, treatment of both inhibitors resulted in suppression of iNOS and COX-2 expressions. Considering roles of MAPKs in inflammatory gene expression, MME inhibited, at least in part, LPS-induced NF-κB activation in the microglial cells by inhibiting the JNK and ERK pathways.

## Conclusions

We have demonstrated that sargachromenol-rich MME inhibits the production of NO, PGE_2_, and pro-inflammatory cytokines as well as iNOS and COX-2 at transcriptional and translational levels. Anti-inflammatory action of MME on LPS-stimulated BV-2 cells was associated with blocking IκB/NF-κB, JNK, and ERK pathways. Verification and confirmation of its anti-inflammatory activity and relative mechanism at the cellular and molecular levels will be beneficial for the further application of MME in therapeutic agents for neuroinflammation in neurodegenerative diseases. Future studies will be necessary in order to determine the bioavailability of this preparation and metabolites in animal tissue.

## Competing interests

The authors declare that they have no competing interests.

## Authors’ contributions

SK carried out the main experiment and wrote the manuscript. MSL prepared MME and performed partial Western blotting. BL contributed to discuss and write the manuscript. EJJ performed immunocytochemistry. WGG assisted ELISA for measuring the proinflammatory cytokines. EJJ and WGG also assisted isolated and identified sargachromenol from MME. NYY collected *M. myogroides*. HRK designed and organized this study. All authors read and approved the final version of the manuscript.

## Pre-publication history

The pre-publication history for this paper can be accessed here:

http://www.biomedcentral.com/1472-6882/14/231/prepub
